# Facile preparation of conductive carbon-based membranes on dielectric substrates

**DOI:** 10.3389/fchem.2023.1152947

**Published:** 2023-03-28

**Authors:** Guoran Liu, Guanchen Xu

**Affiliations:** Advanced Materials Institute, Qilu University of Technology (Shandong Academy of Sciences), Jinan, China

**Keywords:** graphene, CVT, carbon membrane, conductivity, facile preparation

## Abstract

Graphene has attracted much research attention due to its outstanding chemical and physical properties, such as its excellent electronic conductivity, making it as a useful carbon material for a variety of application fields of photoelectric functional devices. Herein, a new method for synthesizing conductive carbon membranes on dielectric substrates *via* a low-temperature thermodynamic driven process is developed. Although the obtained films exhibit low crystallinity, their electrical, wetting, and optical properties are acceptable in practice, which opens up a new avenue for the growth of carbon membranes and may facilitate the applications of transparent electrodes as potential plasma-free surface-enhanced Raman scattering (SERS) substrates.

## 1 Introduction

The preparation of high-quality graphene is challenging and hinders its application in various fields (Chen, et al., 2012; Ren, et al., 2014). Graphene, as a two-dimensional (2D) carbon-based material, has received tremendous scientific attention due to its excellent physical properties, such as a tunable bandgap (Zhang, et al., 2009), extremely high mobility (Geim, 2009; Fang et al., 2015), high mechanical strength (Frank, et al., 2007; Lee, et al., 2008; Vadukumpully, et al., 2011), outstanding light transmittance (Bae et al., 2010; Kim, et al., 2011; Pang, et al., 2011; Liu, et al., 2017), and excellent electronic conductivity (Zhang, et al., 2005; Castro Neto, et al., 2009). Based on these properties, graphene exhibit excellent potential in application of supercapacitors, solar cells, photonic sensors, transparent flexible electrodes, plasma-free surface-enhanced Raman scattering (SERS) substrates, and gene electronic sequencing. As new, exciting characteristics of graphene are continuously being discovered, it has potential applications in many fields. Among all the aforementioned potential applications, transparent electrodes made of graphene are the materials closest to being of practical use (Bae et al., 2010; Kim, et al., 2011; Pang, et al., 2011; Liu et al., 2017). However, it is imperative to develop a reliable synthetic method to make such materials. Graphene can be directly stripped from base material or synthesized on a substrate *via* mechanical exfoliation, direct chemical exfoliation, epitaxial growth, and chemical vapor deposition (CVD) methods. At present, CVD is a promising technique by which to produce high-quality graphene with a large surface area on metal substrates (Li et al., 2009; Reina, et al., 2009; Wei, et al., 2009). For example, in 2009, Li et al. grew a large-area single-layer graphene thin film on a copper substrate *via* CVD using methane (Li et al., 2009). It is generally accepted that the presence of metals is essential for the growth of high-quality graphene as most of the CVD methods reported to date have been based on metal-catalyzed growth. However, an essential and prolix step for such a route is to transport the graphene onto other substrates *via* etching the metal catalyst layer, which may import defects to the as-grown graphene and influence its properties. To date, direct graphene growth on non-conductive substrates *via* CVD has been reported (Fanton et al., 2011; Chen et al., 2014; Chen, et al., 2016; Wang, et al., 2016; Wang, et al., 2019). Although the use of such CVD routes can negate the need for a complex transfer process, the high growth temperature still restricts the choice of substrates and hinders their practical application due to cost. Moreover, the choice of carbon source is restricted to C_x_H_y_ in the gas phase. Therefore, there is a need to find another way to prepare graphene on dielectric substrates.

After decades of continuous development, the chemical vapor transport (CVT) method has shown promising results in solid-phase synthesis, purification, and crystal growth. The basic principles of this technique are that a solid or liquid substance reacts with a catalytic transport agent at a certain temperature to form a gas-phase product, which then migrates through a reactor that has been sealed and evacuated by heating in a tube furnace. This gas-phase reaction product undergoes a reverse reaction in areas of the reactor that are at different temperatures, and, as a result, the gas-phase product is reduced back to the precursor. This process is similar to a sublimation or distillation process. This approach is advantageous because a high-quality, large-area material can be produced in high throughput at a low growth temperature; it has been widely used in the growth of a large number of high-quality single crystals with a layered structure. Hu et al. (2017) used CVT to controllably synthesize 2D MoS_2_, WS_2_, MoSe_2_, and other 2D semiconductors. However, there have been no reports on the preparation of carbon films on dielectric substrates using the CVT method. Therefore, we developed a facile CVT method for the growth of conductive carbon-based membranes using polymers as precursors was developed. Different from other studies (Binnewies, et al., 2013; Ubaldini, et al., 2014; Hu, et al., 2017), the obtained films have an ultra-smooth surface, a continuous structure, and light transmittance and hydrophobic properties, which makes the described CVT method an effective synthesis route for developing a potential chemical mechanism (CM)-based plasma-free SERS platform, electronic sensor devices, materials for energy storage applications, and nanomaterials for use in catalysis (Liang, et al., 2021; Sun, et al., 2021; Liu, et al., 2022; Wang, et al., 2022).

## 2 Results and discussion

**FIGURE 1:**
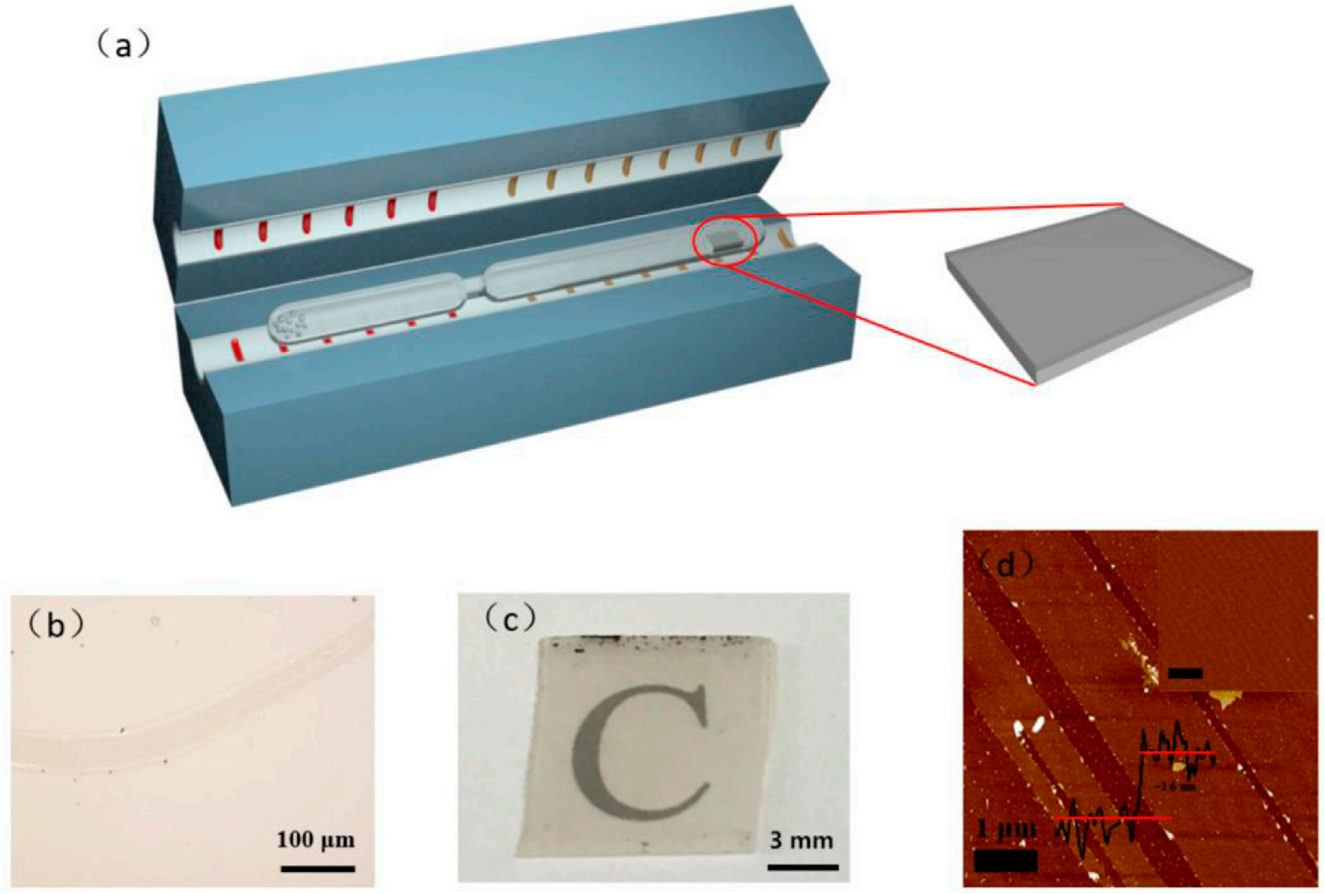
**(A)** Strategy of synthesizing a conductive carbon-based membrane on a dielectric substrate *via* the CVT method. **(B)** Optical micrograph of the carbon membrane. Scale bar, 100 μm. **(C)** An optical image of the carbon membrane. **(D)** AFM images of the carbon membrane.

The synthesis strategy developed in this study is based on the CVT method (see the experimental set-up and details in the ESI). As shown in Figure 1A, a quartz tube with a short and thin column platform was selected as the reaction vessel. First, low density polyethylene (LDPE) was selected as the precursor due to its simple chemical structure, featuring only carbon and hydrogen, and single-side-polished sapphire was selected as a substrate. The LDPE was sealed at one end of a quartz tube and the substrate at the other. Both ends of the closed quartz tube were placed in a horizontal tube furnace. When the precursor end was heated to 850°C, the temperature of the substrate end was 400°C. The polymer precursor was readily cracked into carbon-based fragments under the harsh reaction conditions, which were transported to the substrate end of the reactor *via* a thermodynamic driven process due to the temperature difference between the two ends of the quartz tube. After being annealed at 850°C, the film was deposited across the entire surface of the substrate. Thus, the obtained carbon membrane was transferred *via* a non-etching method.

**FIGURE 2:**
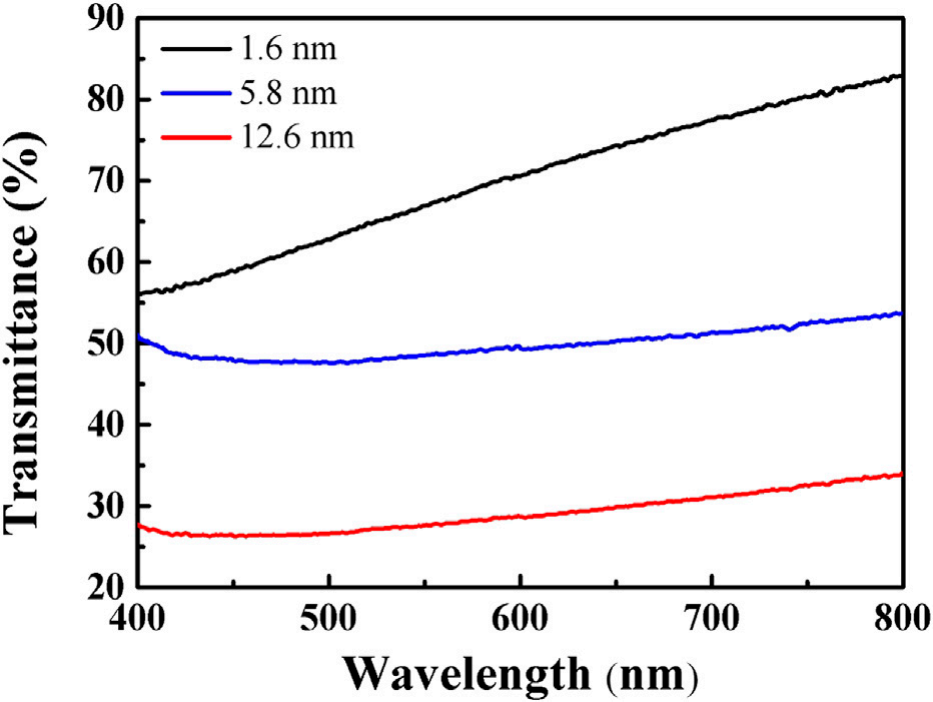
Light transmittance of carbon-based membranes of ∼1.6 nm (black), ∼5.8 nm (blue), and ∼12.6 nm (red) in thickness.

**FIGURE 3:**
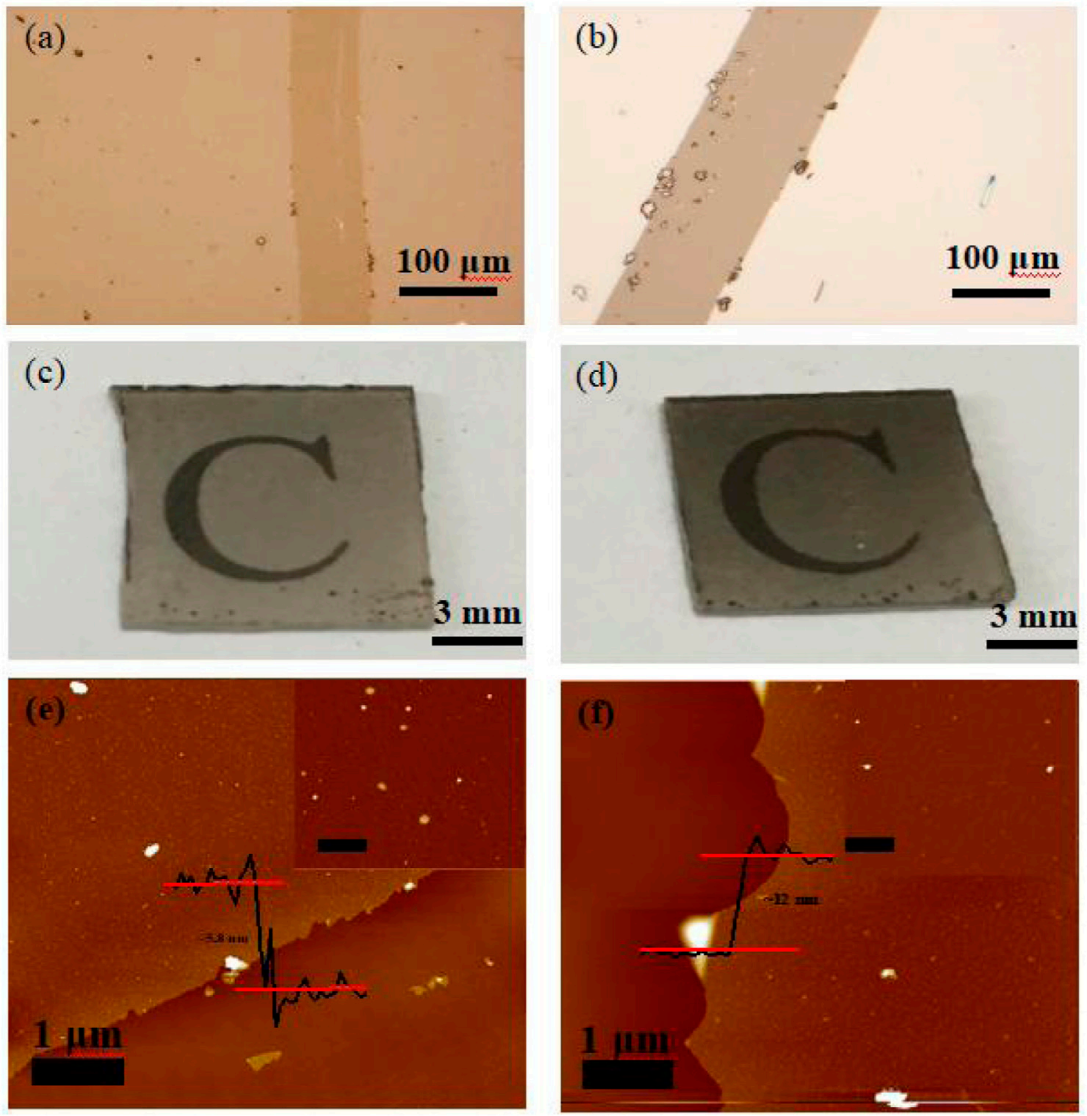
**(A, B)** Optical micrographs of carbon membranes upon an increase in the amount of reactants, **(C, D)** optical images of the carbon membranes. **(E, F)** AFM images of the carbon membrane.

**FIGURE 4:**
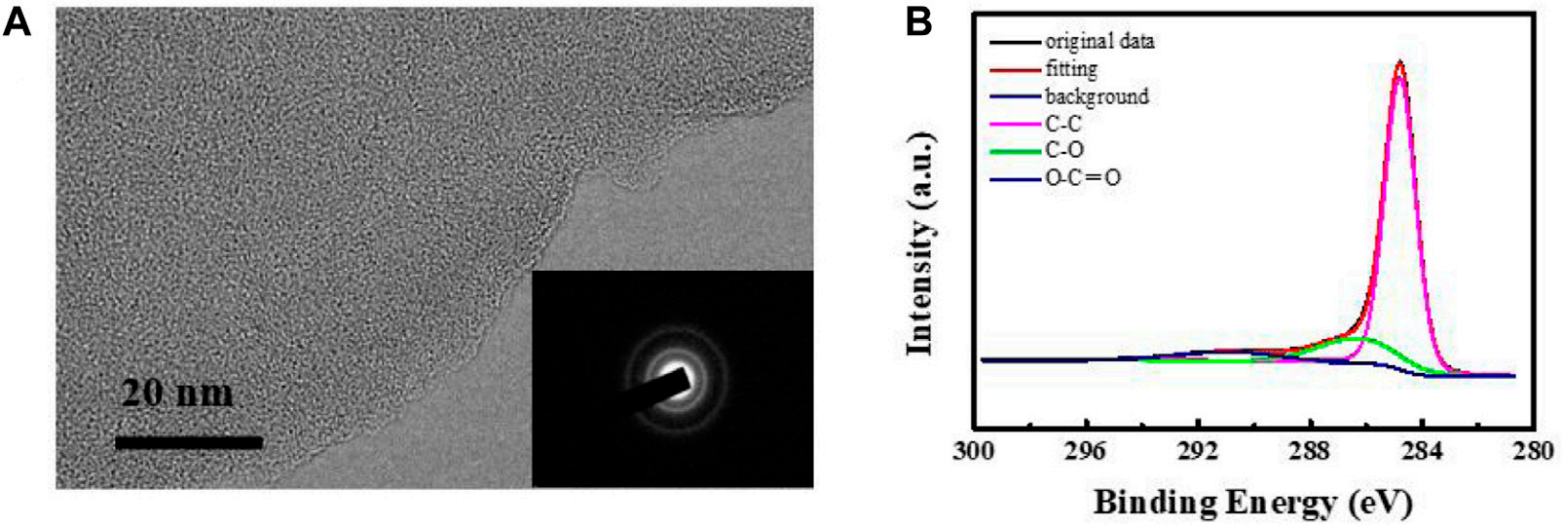
**(A)** TEM image of carbon membrane. **(B)** XPS spectra of carbon membrane.

**FIGURE 5:**
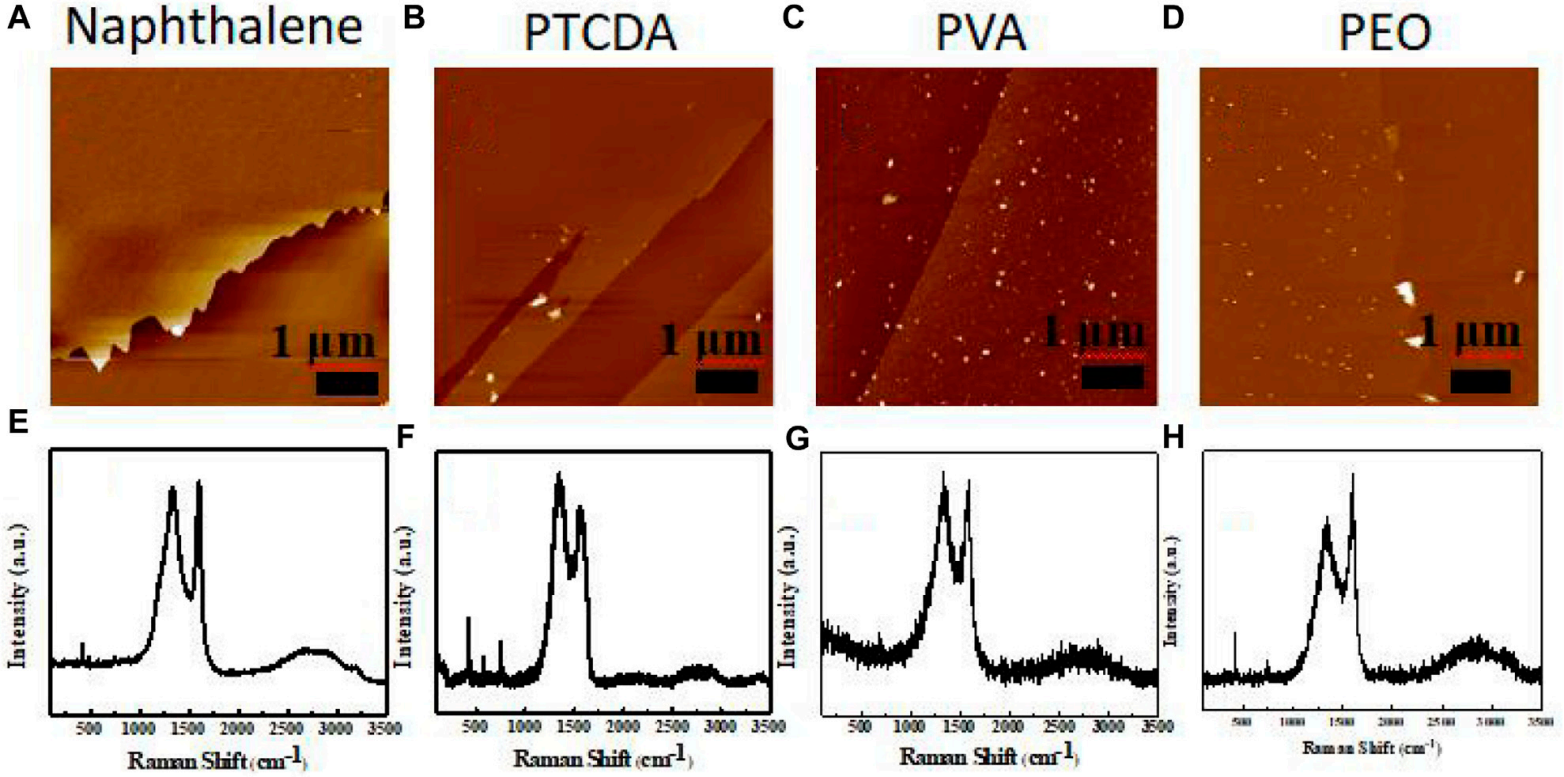
AFM images of carbon membranes prepared using **(A)** naphthalene, **(B)** PTCDA, **(C)** PVA, and **(D)** PEO. Raman spectra of carbon membranes prepared using **(E)** naphthalene, **(F)** PTCDA, **(G)** PVA, and **(H)** PEO.

Based on the optical micrograph shown in Figure 1B, the obtained carbon membrane exhibits a uniform and continuous structure. It can be seen that the film contrast is very close to that of the sapphire substrate through the scratch made using tweezers to distinguish the contrast difference, suggesting that the carbon film was thin enough to exhibit a good transmittance of ∼83% at a wavelength of 800 nm (Figure 1C; Figure 2). Atomic force microscopy (AFM) was used to characterize the thickness of the synthesized membrane. Figure 1D shows an AFM image of the ∼1.6 nm film formed after the CVT process, consistent with the optical image of the same material. It is remarkable that the film surface is smooth compared to that of graphene glass prepared *via* CVD (Binnewies, et al., 2013; Ubaldini, et al., 2014; Hu, et al., 2017), with no vertical and ravine-like product, which can be observed in the inset of Figure 1D. This may be due to the lower reaction temperature and transmission mode of the carbon fragments compared with other methods. The thickness of the film was found to be tunable; it is dependent on the amount of solid carbon precursor used. With an increase in the amount of the reactant, a darker substrate (Figure 3; Figure 4) and a thicker film (Figure 4Sc) were observed upon AFM imaging. In addition to LDPE, there are other precursors that can be employed to make this kind of film, such as naphthalene, polyvinyl alcohol (PVA), polyethylene oxide (PEO), and perylene-3,4,9,10-tetracarboxylic dianhydride (PTCDA)*.* Using these precursors, carbon films with similar properties were obtained under the same experimental conditions (Figures 5A–D).

The microstructure of the film prepared by CVT was probed by transmission electron microscopy (TEM), selected-area electron diffraction (SAED), and X-ray photoelectron spectroscopy (XPS). The TEM image (Figure 4A) shows that the carbon membrane exhibits a flat surface with no crystal lattice, confirming that an amorphous phase was formed during the CVT process. The SAED pattern exhibits typical amorphous carbon rings without any diffraction dots, in agreement with the TEM results. The results thus reflect that a complex cracking process of LDPE occurred upon an increase in temperature (Aboulkas, et al., 2010). To further investigate the structure of the prepared carbon film, more detailed information about its chemical composition was obtained from the C 1s XPS spectrum (Figure 4B). The deconvoluted spectrum features peaks at binding energies of 284.8 eV, 286.4 eV, and 290.7 eV, attributed to C–C, C–O and O–C=O bonds, respectively. This confirmed the presence of a large number of oxygen-containing functional groups in the prepared films. Amorphous carbon is an amorphous metastable material composed of sp^2^ and sp^3^ hybrid carbon atoms. The C 1s nuclear binding energy (284.7 eV) of the thin film is close to the value of diamond (285.3 eV), indicating that it contains a high amount of sp^3^ hybridized carbon. The results thus support the formation of a graphitized conductive carbon film.

**FIGURE 6:**
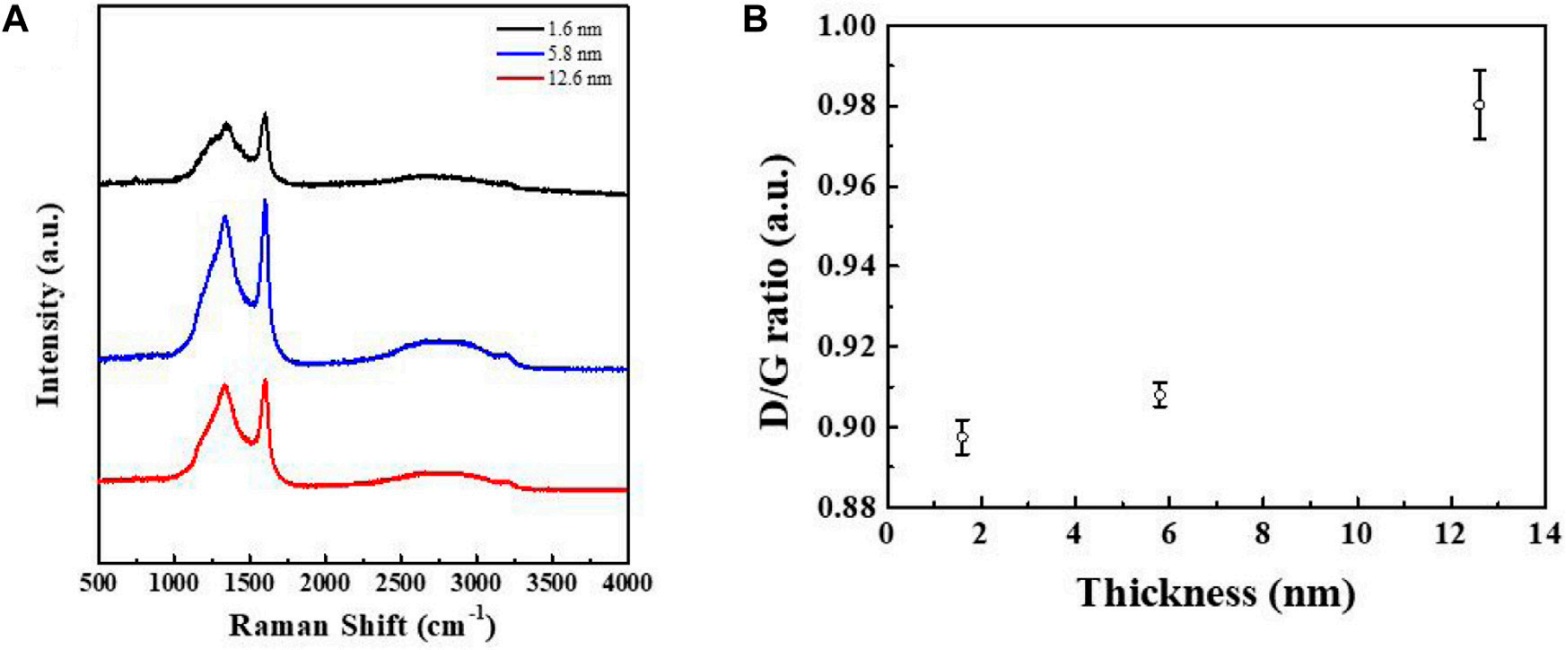
**(A)** Raman spectra of carbon films with different thicknesses. **(B)** Relationship between the ratio of the D peak to G peak and the thickness of the carbon film.

Raman spectroscopy is a very effective structural characterization method for carbon materials, which is used to determine their microstructure *via* different vibration modes and strengths. To confirm the quality of this film, the Raman spectra of three films with different thicknesses are shown in Figure 6A, wherein it can be seen that the films of different thickness all feature two peaks in the same positions. The two broad peaks at around 1,341 cm^−1^ and 1,594 cm^−1^ can be attributed to the characteristic peaks of the D and G bands of carbon, corresponding to the sp^3^ hybridization of carbon with a disordered structure and the sp^2^ hybridization of a graphitized structure, respectively (Ferrari et al., 2006; Ferrari, 2007). In addition, upon an increase in film thickness, the positions of the two peaks remained almost unchanged. The results show the presence of amorphous carbon in the film, which is also consistent with the SAED results. Figure 6B shows that the intensity ratio of the D peak (*I*_D_) with respect to the G peak (*I*_G_) increases in line with an increase in film thickness. The ratio of the two peak strengths is an important criterion by which to judge the degree of graphitization of carbon materials. The smaller the ratio, the less amorphous the carbon. Compared with LDPE, the conductive carbon films prepared using the other polymers as reactants were disordered, and only the product prepared using PEO as a precursor showed a high degree of graphitization (Figures 5E–H). This indicates that this method of preparing conductive carbon films has great application value, thus providing ideas for further research.

**FIGURE 7:**
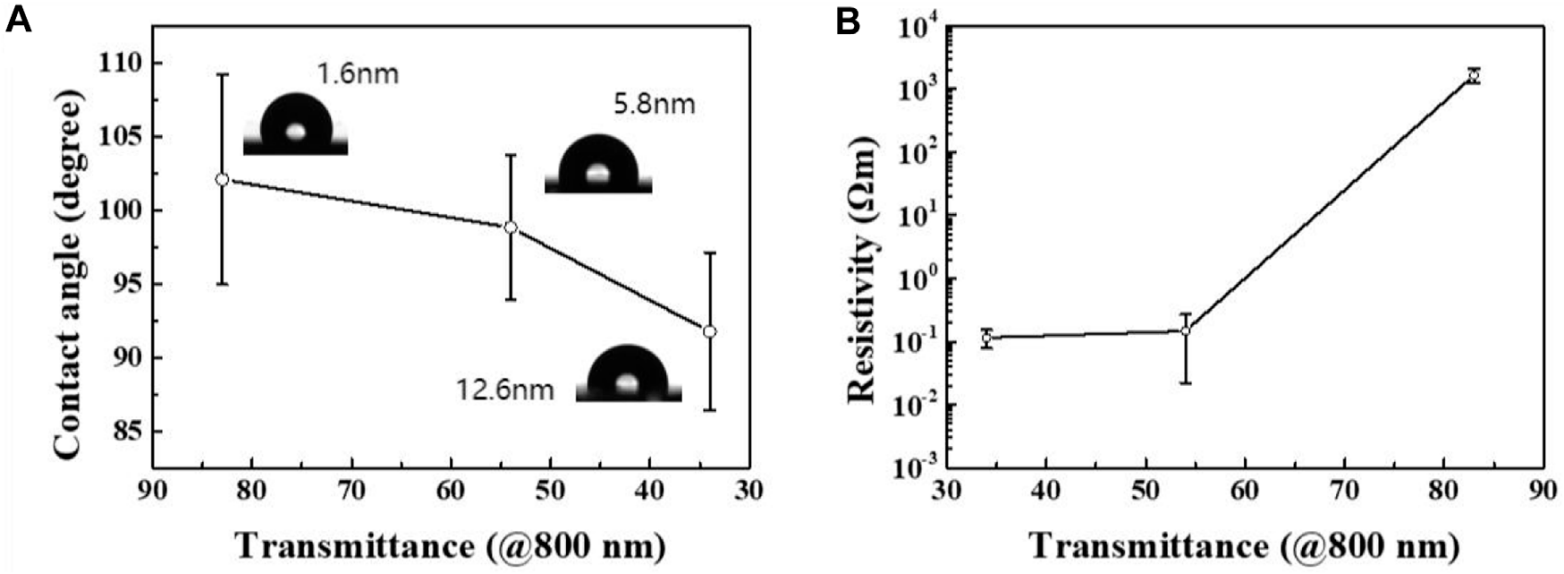
**(A)** Relationship between the contact angle of the carbon film and the light transmittance at a wavelength of 800 nm. **(B)** Relationship between the resistivity of the carbon film and the light transmittance at a wavelength of 800 nm.

The properties of the films were investigated and the results are displayed in Figure 7. As shown in Figure 7A, at a wavelength of 800 nm, for ∼1.6 nm, ∼5.8 nm, and ∼12.6 nm films with different light transmittance (Figure 2) the average contact angles were 102°, 99°, and 92°, respectively. Thicker films with low light transmittance exhibited smaller contact angles and better hydrophilicity. The presence of peaks related to ester groups in the XPS data also provided evidence that upon an increase in thickness the O−C=O bonding increased and the films became more hydrophilic. The results of conductivity measurements of films prepared at different transmittance were as expected, as shown in Figure 7B. It can be seen that the resistivity increases with the thickness of the film. The bonding between carbon atoms in the thin film is mainly related to sp^2^ and sp^3^ hybridization, among which the former carbon chain structure has good conductivity, while the latter carbon chain structure has poor conductivity. By controlling the reversible transformation between carbon atoms and hybrid binding modes using an external electric field, the reversible transformation between the high and low resistance of the carbon film can be controlled and the electric resistance effect can be realized. The thin film prepared on the dielectric plate can thus be applied without transfer and etching, which has good prospects for electrical applications.

## 3 Materials and methods

LDPE, naphthalene, PVA, PEO, and PTCDA were purchased from Alfa Aesar. The quartz reaction tube was soaked in potassium hydroxide solution to remove organic impurities and was washed with deionized water before use.

### 3.1 Synthesis of the carbon membrane using LDPE

LDPE (1.5 mg) was added into one end of a 1-cm quartz tube with a 0.5-cm column platform of 0.5 cm in length, and then the sapphire substrate was placed inside. A CH_4_–O_2_ flame and a vacuum system were used to cut off and seal the quartz tube. The precursor was subjected to a temperature of 850°C to achieve a temperature of 400°C at which to form the product, with the final product obtained after annealing.

### 3.2 Characterization

Optical images were captured using an Olympus BX 53M microscope. AFM images were taken using a Bruker Bioscope Resolve in ScanAsyst. Raman and photoluminescence (PL) measurements were conducted using a Horiba-Smart Raman system at 532 nm laser excitation at a power of 0.5 mW. The Si peak at 520.7 cm^−1^ was used for calibration in the data analysis of Raman and PL spectra. TEM images and SAED patterns were acquired using an F200 s instrument at 200 kV. XPS measurements were conducted on a Thermo ESCALAB 250XI spectrometer. Contact angles were measured and calculated using a DSA100E system. Transmittance measurements were conducted using an LS116 light transmittance instrument. Resistivity measurements were conducted using a four-electrode resistance measuring meter.

## 4 Conclusion

In summary, a novel and effective method is firstly developed for synthesizing conductive carbon membranes on dielectric substrates. A carbon film can be directly synthesized on a dielectric substrate, which can then be directly incorporated into electronic devices, thus avoiding a complex and post-synthetic transfer process that may lead to contamination and breakage of the film. Due to the fragmentation of LDPE is able to occur in many different ways, the structure of the thin film prepared using the developed CVT method was amorphous, and its thickness was controllable. The conductivity of the film was found to be related to the degree of deposition of the LDPE andthe use of different reactants led to the same results, further illustrating the extensibility and convenience of this experimental method. These characteristics make the carbon films prepared *via* this method ideal materials that have broad application potential for use in photoelectric chemistry, as potential CM-based plasma-free SERS platforms, transparent flexible electrodes, supercapacitors, and solar cells in the energy storage fields.

## Data Availability

The original contributions presented in the study are included in the article/Supplementary Materials, further inquiries can be directed to the corresponding author.

## References

[B1] Aboulkas A. El harfi K. El Bouadili A. (2010). Thermal degradation behaviors of polyethylene and polypropylene. Part i: Pyrolysis kinetics and mechanisms. Energy Convers. Manag. 51, 1363–1369. 10.1016/j.enconman.2009.12.017

[B2] Bae S. Kim H. Lee Y. Xu X. Park J. S. Zheng Y. (2010). Roll-to-roll production of 30-inch graphene films for transparent electrodes. Nat. Nanotechnol. 5, 574–578. 10.1038/nnano.2010.132 20562870

[B3] Binnewies M. Glaum R. Schmidt M. Schmidt P. (2013). Chemical vapor transport reactions - a historical review. Z. Anorg. Allg. Chem. 639, 219–229. 10.1002/zaac.201300048

[B4] Castro Neto A. H. Guinea F. Peres N. M. R. Novoselov K. S. Geim A. K. (2009). The electronic properties of graphene. RMP 81, 109–162. 10.1103/revmodphys.81.109

[B5] Chen J. Y. Guo Y. Jiang L. Xu Z. Huang L. Xue Y. (2014). Near-equilibrium chemical vapor deposition of high-quality single-crystal graphene directly on various dielectric substrates. Adv. Mat. 26, 1348–1353. 10.1002/adma.201304872 24338972

[B6] Chen L. Hernandez Y. Feng X. Mullen K. (2012). From nanographene and graphene nanoribbons to graphene sheets: Chemical synthesis. Angew. Chem. Int. Ed. 51, 7640–7654. 10.1002/anie.201201084 22777811

[B7] Chen X. Wu B. Liu Y. (2016). Direct preparation of high quality graphene on dielectric substrates. Chem. Soc. Rev. 45, 2057–2074. 10.1039/c5cs00542f 26847929

[B8] Fang X. Y. Yu X. X. Zheng H. M. Jin H. B. Wang L. Cao M. S. (2015). Temperature- and thickness-dependent electrical conductivity of few-layer graphene and graphene nanosheets. Phys. Lett. A 379, 2245–2251. 10.1016/j.physleta.2015.06.063

[B9] Fanton M. A. Robinson J. A. Puls C. Liu Y. Hollander M. J. Weiland B. E. (2011). Characterization of graphene films and transistors grown on sapphire by metal-free chemical vapor deposition. ACS Nano 5, 8062–8069. 10.1021/nn202643t 21905713

[B10] Ferrari A. C. Meyer J. C. Scardaci V. Casiraghi C. Lazzeri M. Mauri F. (2006). Raman spectrum of graphene and graphene layers. Phys. Rev. Lett. 97, 187401. 10.1103/physrevlett.97.187401 17155573

[B11] Ferrari A. C. (2007). Raman spectroscopy of graphene and graphite: Disorder, electron-phonon coupling, doping and nonadiabatic effects. Solid State Commun. 143, 47–57. 10.1016/j.ssc.2007.03.052

[B12] Frank I. W. Tanenbaum D. M. van der Zande A. M. McEuen P. L. (2007). Mechanical properties of suspended graphene sheets. J. Vac. Sci. Technol. 25, 2558–2561. 10.1116/1.2789446

[B13] Geim A. K. (2009). Graphene: Status and prospects. Science 324, 1530–1534. 10.1126/science.1158877 19541989

[B14] Hu D. K. Xu G. Xing L. Yan X. Wang J. Zheng J. (2017). Two-dimensional semiconductors grown by chemical vapor transport. Angew. Chem. Int. Ed. 56, 3665–3669. 10.1002/ange.201700439 28220992

[B15] Kim K. S. Zhao Y. Jang H. Lee S. Y. Kim J. M. Kim K. S. (2009). Large-scale pattern growth of graphene films for stretchable transparent electrodes. Nature 457, 706–710. 10.1038/nature07719 19145232

[B16] Lee C. Wei X. Kysar J. W. Hone J. (2008). Measurement of the elastic properties and intrinsic strength of monolayer graphene. Science 321, 385–388. 10.1126/science.1157996 18635798

[B17] Li X. S. Cai W. An J. Kim S. Nah J. Yang D. (2009). Large-area synthesis of high-quality and uniform graphene films on copper foils. Science 324, 1312–1314. 10.1126/science.1171245 19423775

[B18] Liang X. Li N. Zhang R. Yin P. Zhang C. Yang N. (2021). Carbon-based SERS biosensor: From substrate design to sensing and bioapplication. NPG Asia Mater 13, 8–36. 10.1038/s41427-020-00278-5

[B19] Liu G. Mu Z. Guo J. Shan K. Shang X. Yu J. (2022). Surface-enhanced Raman scattering as a potential strategy for wearable flexible sensing and point-of-care testing non-invasive medical diagnosis. Front. Chem. 10, 1060322. 10.3389/fchem.2022.1060322 36405318 PMC9669362

[B20] Liu N. Chortos A. Lei T. Jin L. Kim T. R. Bae W. G. (2017). Ultratransparent and stretchable graphene electrodes. Sci. Adv. 3, e1700159. 10.1126/sciadv.1700159 28913422 PMC5590784

[B21] Pang S. P. Hernandez Y. Feng X. Mullen K. (2011). Graphene as transparent electrode material for organic electronics. Adv. Mat. 23, 2779–2795. 10.1002/adma.201100304 21520463

[B22] Reina A. Jia X. Ho J. Nezich D. Son H. Bulovic V. (2009). Large area, few-layer graphene films on arbitrary substrates by chemical vapor deposition. Nano Lett. 9, 30–35. 10.1021/nl801827v 19046078

[B23] Ren W. C. Cheng H. M. (2014). The global growth of graphene. Nat. Nanotechnol. 9, 726–730. 10.1038/nnano.2014.229 25286256

[B24] Sun G. Li N. Wang D. Xu G. Zhang X. Gong H. (2021). A novel 3D hierarchical plasmonic functional Cu@Co_3_O_4_@Ag array as intelligent SERS sensing platform with trace droplet rapid detection ability for pesticide residue detection on fruits and vegetables. Nanomaterials 11, 3460–3474. 10.3390/nano11123460 34947808 PMC8705477

[B25] Ubaldini A. Giannini E. (2014). Improved chemical vapor transport growth of transition metal dichalcogenides. J. Cryst. Growth. 401, 878–882. 10.1016/j.jcrysgro.2013.12.070

[B26] Vadukumpully S. Paul J. Mahanta N. Valiyaveettil S. (2011). Flexible conductive graphene/poly(vinyl chloride) composite thin films with high mechanical strength and thermal stability. Carbon 49, 198–205. 10.1016/j.carbon.2010.09.004

[B27] Wang D. Xu G. Zhang X. Gong H. Jiang L. Sun G. (2022). Dual-functional ultrathin wearable 3D particle-in-cavity SF–AAO–Au SERS sensors for effective sweat glucose and lab-on-glove pesticide detection. Sens. Actuators B Chem. 359, 131512. 10.1016/j.snb.2022.131512

[B28] Wang H. P. Xue X. Jiang Q. Wang Y. Geng D. Cai L. (2019). Primary nucleation-dominated chemical vapor deposition growth for uniform graphene monolayers on dielectric substrate. J. Am. Chem. Soc. 141, 11004–11008. 10.1021/jacs.9b05705 31265267

[B29] Wang H. P. Yu G. (2016). Direct CVD graphene growth on semiconductors and dielectrics for transfer-free device fabrication. Adv. Mat. 28, 4956–4975. 10.1002/adma.201505123 27122247

[B30] Wei D. C. Liu Y. Wang Y. Zhang H. Huang L. Yu G. (2009). Synthesis of n-doped graphene by chemical vapor deposition and its electrical properties. Nano Lett. 9, 1752–1758. 10.1021/nl803279t 19326921

[B31] Zhang Y. B. Tan Y. W. Stormer H. L. Kim P. (2005). Experimental observation of the quantum hall effect and berry's phase in graphene. Nature 438, 201–204. 10.1038/nature04235 16281031

[B32] Zhang Y. B. Tang T. T. Girit C. Hao Z. Martin M. C. Zettl A. (2009). Direct observation of a widely tunable bandgap in bilayer graphene. Nature 459, 820–823. 10.1038/nature08105 19516337

